# Prognostic value of circulating microRNA-21-5p and microRNA-126 in patients with acute myocardial infarction and infarct-related artery total occlusion

**DOI:** 10.3389/fcvm.2022.947721

**Published:** 2022-10-18

**Authors:** Xiao-long Mi, Yu-ping Gao, Da-jie Hao, Zhi-jun Zhang, Zheng Xu, Tong Li, Xue-wen Li

**Affiliations:** Department of Cardiovascular Medicine, Shanxi Bethune Hospital, Tongji Shanxi Hospital, Third Hospital of Shanxi Medical University, Shanxi Academy of Medical Sciences, Taiyuan, China

**Keywords:** circulating miRNA, acute myocardial infarction, biomarker, microRNA-21-5p, microRNA-126

## Abstract

**Background:**

Cardiovascular disease, including acute myocardial infarction (AMI), is a major global cause of mortality and morbidity. Specificity and sensitivity limit the utility of classic diagnostic biomarkers for AMI. Therefore, it is critical to identify novel biomarkers for its accurate diagnosis. Cumulative studies have demonstrated that circulating microRNAs (miRs) participate in the pathophysiological processes of AMI and are promising diagnostic biomarkers for the condition. This study aimed to ascertain the diagnostic accuracy of circulating miR-21-5p and miR-126 used as biomarkers in patients with AMI and infarct-related artery total occlusion (IR-ATO) or infarct-related blood-vessel recanalization (IR-BVR).

**Methods:**

The expression of miR-21-5p and miR-126 was examined separately in 50 healthy subjects, 51 patients with IR-ATO AMI, and 49 patients with IR-BVR AMI using quantitative real-time polymerase chain reaction.

**Results:**

When compared with the control group, the IR-ATO AMI group exhibited increased miR-21-5p (*p* < 0.0001) and miR-126 (*p* < 0.0001), and the IR-BVR AMI group exhibited increased miR-21-5p (*p* < 0.0001). However, there was no significant difference in miR-126 between the IR-BVR AMI and the control groups. A Spearman's correlation coefficient showed a strong correlation was found between miR-21-5p, miR-126, cardiac troponin-I, and creatine kinase isoenzyme in all three groups, while a receiver operating characteristic analysis revealed that miR-21-5p and miR-126 exhibited considerable diagnostic accuracy for IR-ATO AMI.

**Conclusion:**

Circulating miR-21-5p and miR-126 may be promising prognostic biomarkers for patients with AMI and IR-ATO.

## Introduction

Cardiovascular disease is the leading cause of death worldwide, and coronary artery disease (CAD) accounts for half of those deaths (1). In CAD, acute myocardial infarction (AMI) caused by the occlusion of the coronary arteries and leading to the cell necrosis of cardiomyocytes plays a major role in acute and chronic heart failure (2). The accurate diagnosis of AMI is an unmet clinical need that could help to reduce the incidence of heart failure after AMI (3). Currently, the diagnosis of AMI depends on at least two of the following detailed clinical features: Q waves in electrocardiograms (ECGs), the elevation of or decrease in cardiac markers, e.g., creatine kinase isoenzyme (CK-MB), and increased levels of serum cardiac troponin (cTn) -I and -T alone or in combination (4–6). However, CK-MB lacks sensitivity for the onset of AMI, especially within 6 h (7). In addition, elevated cTn occurs not only in patients with heart failure but also in those with chronic kidney diseases (8). Therefore, it is critical to identify sensitive and specific biomarkers for the accurate diagnosis of AMI.

MicroRNA (miR), endogenous short (20–22 nucleotides) non-coding RNA, has been shown to participate in diverse cellular processes, including cancer, cardiovascular diseases, and inflammatory diseases (9–11). The miRs perform physiological and pathological functions by regulating the expression of target genes (12). Among them, miR-21, identified initially as a tumor growth enhancer, has been shown to participate in the initiation and progression of AMI (13). Published studies found that the upregulation of miR-21 or phosphatase and tensin homolog (PTEN) silencing improved cardiomyocytes' viability and reduced the apoptotic cell rate by activating the phosphatidylinositol 3-kinase/serine/threonine kinase signaling pathway (14), and the overexpression of miR-21 reduced the infarct size and immune cell infiltration in a rat model with AMI (15). However, miR-126, which is highly enriched with endothelial cells, has been observed to increase in the plasma or serum of patients with AMI from the onset of the condition (16). Therefore, miR-21 and miR-126 are considered promising biomarkers for the diagnosis of AMI.

In the present study, we detected the expression of circulating miR-21-5p and miR-126 in patients with AMI and infarct-related artery total occlusion (IR-ATO) or infarct-related blood-vessel recanalization (IR-BVR). We looked for correlations between miRs, CK-MB, and cTn-I to investigate the sensitivity and reliability of these biomarkers in identifying AMI and its possible clinical type.

## Methods

### Study subjects

A total of 100 consecutive patients, aged 26–87 y (54 ± 12 y), with AMI and ST-segment elevation myocardial infarction (STEMI) were enrolled in this case-controlled study from January 2018 to January 2019.

The inclusion criteria were as follows: (1) persistent chest pain for ≥30 min, (2) significant ST-segment elevation (≥0.1 or ≥0.2 mV on ≥2 adjacent limb or precordial leads, respectively, or new left bundle-branch block), or (3) the onset of chest pain within 12 h or after more than 12 h accompanied by hemodynamic instability and hypovolemic shock.

The exclusion criteria were as follows: thrombolytic therapy for STEMI, valvular heart disease, myocarditis, cardiomyopathy, severe heart failure, severe liver and kidney dysfunction, hematological system diseases, malignant tumors, gastrectomy, acute and chronic infectious diseases, autoimmune diseases, bronchus asthma, abnormal calcium metabolism, thyroid or adrenal dysfunction, history of surgery, or trauma.

The patients were divided into the IR-ATO group (*n* = 51) and the IR-BVR group (*n* = 49) according to their coronary angiography results. In addition, 50 healthy patients without a history of heart, vascular, or other major disease and with a normal chest X-ray, ECG, liver and kidney function, and biochemical index were recruited as the control group. The baseline characteristics of all subjects were collected.

The Gensini score was used to evaluate the degree of coronary artery disease in patients, and the correlations between the Gensini score and the levels of miR-21-5p and miR-126 were analyzed. The Gensini scoring criteria were as follows: 1 point: stenosis <25%; 2 points: stenosis 25–49%, 4 points, stenosis 50–74%; 8 points, 75–89% stenosis; 16 points, 90–99% stenosis; 32 points, 100% stenosis in the lesion.

This study was approved by the ethics committee of the Shanxi Bethune Hospital, and all participants signed an informed consent form.

### Sample collection

Approximately 10 ml of venous blood was collected from each participant in ethylene diamine tetra acetic acid-coated tubes. All samples were centrifuged at 3,000 rpm for 10 min at 4°C. The supernatant was removed and saved at −20°C for future use.

### Measurement of cardiac troponin-I and creatine kinase isoenzyme

According to the manufacturer's protocol, the serum cTn-I concentration and CK-MB activity were measured using an enzyme-linked immunosorbent assay kit and immunoassay analyzer, respectively.

### Total RNA extraction and microRNA reverse transcription

Total RNA was extracted from serum using TRNzol Universal Reagent (TIANGEN^®^ Biotech, Beijing, China) based on the protocol provided by the manufacturer. Serum samples (0.25 ml) were homogenized in TRIzol™ (0.75 ml) and stored at room temperature for 5 min. Chloroform was added to each sample (0.2 ml) before it was shaken vigorously to ensure complete dissociation of the nucleoprotein complexes. After standing at room temperature for 10 min, the mixtures were centrifuged at 12,000 × g for 15 min at 4°C. The RNA in the aqueous phase was precipitated with cold isopropyl alcohol (0.5 ml). Following centrifugation at 12,000 × g for 15 min, the pellets, i.e., RNA, were washed with 75% ethanol and finally dissolved with diethyl pyrocarbonate H_2_O (10 μl). The RNA purity was determined using a NanoDrop™ 2000 RNA analyzer (Thermo Fisher Scientific, Waltham, MA, USA), and only those samples with a ratio of between 1.8 and 2.1 were used in the present study.

Reverse transcription was performed using 1 μg of total RNA in a total reaction volume of 20 μl using a First Strand cDNA Synthesis kit (KR201, TIANGEN^®^). The 20-μl reactions were incubated at 37°C for 1 h and terminated by heating to 85°C for 5 min. Following cDNA synthesis, all cDNA samples were stored at −20°C.

### MicroRNA quantitative real-time polymerase chain reaction

A quantitative real-time polymerase chain reaction (qPCR) was performed using a real-time PCR kit (Takara, Beijing, China) and an Applied Biosystems™ 7500 real-time PCR system (Thermo Fisher Scientific, Inc.). The sequences of all the primers used in our study are shown in Table 1. For normalization, U6 small nuclear RNA was used as an endogenous control. The 20-μl qPCR reactions were incubated at 95°C for 30 s followed by 40 cycles of 95°C for 4 s and 60°C for 40 s. The data were processed using the relative quantification method and calculated using the 2^−ΔΔ*Ct*^ method. There were 10 replicates for each group.

**Table 1:** Primer sequence.

**Primer**	**Sequence (5^′^to 3^′^)**
miR-21-5p forward	TAGCTTATCAGACTGATGTTG
miR-21-5p reverse	GTGCGTGTCGTGGAGTCG
miR-126 forward	TCGTACCGTGAGTAATAATGCG
miR-126 reverse	GTGCGTGTCGTGGAGTCG
U6 forward	CTCGCTTCGGCAGCACA
U6 reverse	AACGCTTCACGAATTTGCGT

### Data analysis and statistics

For statistical analyses, the GraphPad Prism 6.0 software package (GraphPad Software, Inc., La Jolla, CA, USA) was utilized to visualize the data. The normality of the data was tested by the Kolmogorov–Smirnov test and Shapiro–Wilk test. Normally distributed data were presented as mean ± SD and were compared using a Student's *t*-test. The expressions of miR-21-5p and miR-126 were compared between groups using a one-way analysis of variance followed by Tukey's test. The Spearman's rank correlation coefficient was used to evaluate the association between miR levels and between these levels and cTn-I and CK-MB. Pearson's correlation analysis was used to analyze the correlations of the Gensini score with the plasma levels of miR-21-5p and miR-126.

Logistic regression was used to correct for confounding factors. The receiver operating characteristic (ROC) and area under the curve (AUC) were calculated to evaluate the diagnostic accuracy of miR-21-5p and miR-126. All statistical tests were two-tailed, and *p* < 0.05 was considered statistically significant.

## Results

### Baseline clinical characteristics of the study subjects

The baseline clinical characteristics of all the study subjects are listed in Table 2. Statistically significant differences existed in the values for body mass index (BMI), systolic blood pressure (SBP), diastolic blood pressure (DBP), low-density lipoprotein cholesterol (LDL-C), blood glucose (GLU), creatinine (CRE), and left ventricular ejection fraction (LVEF) between the control group and the IR-ATO and IR-BVR groups. When compared with the control group, BMI, SBP, DBP, triglyceride (TG), total cholesterol (TC), LDL-C, GLU, and CRE were higher, while LDL-C, N-terminal prohormone of brain natriuretic peptide (NT-proBNP), and LVEF were lower in the IR-ATO and the IR-BVR groups.

**Table 2:** Baseline clinical characteristics of the study subjects.

**Parameters**	**Controls (*n =* 50)**	**Subjects with VTO (*n =* 51)**	**Subjects with VIO (*n =* 49)**
BMI	21.160 ± 0.187	22.670 ± 0.344**	23.080 ± 0.279**
SBP	114.800 ± 1.512	135.000 ± 3.687**	129.900 ± 2.474**
DBP	73.080 ± 0.849	83.290 ± 1.858**	84.840 ± 1.898**
TG (mmol/L)	1.615 ± 0.079	1.498 ± 0.060	1.627 ± 0.072
TC (mmol/L)	4.461 ± 0.144	4.400 ± 0.135	4.732 ± 0.178
LDL-C (mmol/L)	2.749 ± 0.136	2.326 ± 0.051**	2.473 ± 0.080
blood glucose (mmol/L)	5.288 ± 0.138	6.839 ± 0.392**	7.841 ± 0.464**
Creatinine (umol/L)	77.950 ± 2.827	97.270 ± 2.789**	93.800 ± 3.000**
NT-proBNP	764.500 ± 12.230	749.400 ± 41.260	718.600 ± 35.040
LVEF (%)	61.560 ± 0.703	53.760 ± 0.814**	53.020 ± 0.888
MACE (%)	0.000	17.647*	6.122

VTO, vascular total occlusion group; VIO, vascular incomplete occlusion group.

Data are means ± SD.

Student's t-test for the difference between VTO or VIO and the control group. *p < 0.05; **p < 0.001.

### Serum levels of cardiac troponin-I and creatine kinase isoenzyme

The serum levels of cTn-I and CK-MB are shown in Table 3. The average levels of cTn-I and CK-MB were 85.22 ± 8.283 and 86.97 ± 5.984 IU/L, respectively, in the IR-ATO group and 156.5 ± 12.96 and 130.5 ± 5.224 IU/L, respectively, in the IR-BVR group. These levels were significantly higher than 0.1638 ± 0.01549 and 11.19 ± 0.5617 IU/L, respectively, in the control group.

**Table 3:** The expression levels of cTnI and CK-MB.

**Parameters**	**Control (*n* = 50)**	**VTO (*n* = 51)**	**VIO (*n* = 49)**
cTnI (IU/L)	0.1638 ± 0.01549	85.22 ± 8.283**	86.97 ± 5.984**
CK-MB (IU/L)	11.19 ± 0.5617	156.5 ± 12.96**	130.5 ± 5.224**

VTO, vascular total occlusion group; VIO, vascular incomplete occlusion group.

Data are means ± SD.

Student's t-test for the difference between VTO or VIO and the control group.

**p < 0.001.

### Expression levels of miR-21-5p and miR-126

**Figure 1:**
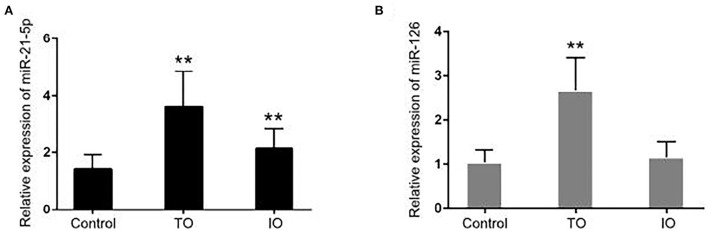
Expressions of miR-21-5p and miR-126 in all subjects. The bar chart shows the expressions of **(A)** miR-21-5p and **(B)** miR-126 measured by quantitative real-time polymerase chain reaction in the control group (*n* = 50), infarct-related artery total occlusion (IR-ATO) acute myocardial infarction (AMI) group (*n* = 51), and IR-blood-vessel recanalization (BVR) AMI group (*n* = 49). The relative microRNA expression was calculated using the 2^−ΔΔ*Ct*^ method. The differences between the groups were compared using a one-way analysis of variance (**p < 0.001). The IR-ATO group is denoted by VO, and the IR-BVR group is denoted by VIO.

The differences in the expression of miR-21-5p and miR-126 among the groups are displayed in Figure 1. There was a significant difference in the relative expression of miR-21-5p between the IR-ATO group (3.616 ± 0.1718) and the control group (1.431 ± 0.06963) and between the IR-BVR group (2.126 ± 0.1026) and the control group. In addition, the relative expression of miR-126 in the vascular group (i.e., the IR-ATO and IR-BVR groups) of 2.667 ± 0.1045 was significantly higher than in the control group (1.04 ± 0.04034). However, there was no difference in the relative expression of miR-126 between the IR-BVR (1.16 ± 0.0496) and the control groups.

**Figure 2:**
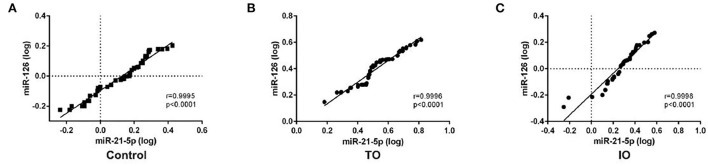
Spearman's correlation coefficient between circulating miR-21-5p and miR-126 in all subjects. The scatter plots show the strong correlation between miR-21-5p and miR-126 in the control group (*n* = 50), **(A)** infarct-related artery total occlusion (IR-ATO) acute myocardial infarction (AMI) group (*n* = 51), **(B)** IR blood-vessel recanalization (BVR) AMI group (*n* = 49), and **(C)** VO = IR-ATO group, and VIO = IR-BVR group.

Spearman's correlation coefficient was calculated to ascertain whether the expressions of miR-21-5p and miR-126 were correlated. The Spearman's correlation coefficient between miR-21-5p and miR-126 in the IR-ATO, IR-BVR, and control groups was 0.9995 (*p* < 0.0001), 0.9998 (*p* < 0.0001), and 0.9996 (*p* < 0.0001), respectively (Figure 2).

### Correlation analysis

The Spearman's correlation coefficient was also calculated to ascertain the correlation between miR-21-5p and cTn-I, miR-21-5p and CK-MB, miR-126 and cTn-I, and miR-126 and CK-MB (see Table 4). A strong correlation existed between miR-21-5p and cTn-I or CK-MB and between miR-126 and cTn-I or CK-MB in all groups.

**Table 4:** Spearman correlation between miR-21-5p, miR-126, cTnI, and CK-MB.

**Parameters**	**Control**		**VTO**		**VIO**	
	**miR-21-5p**	**miR-126**	**miR-21-5p**	**miR-126**	**miR-21-5p**	**miR-126**
cTnI	r = 0.9984	r = 0.9984	r = 0.9998	r = 0.9997	r = 0.9999	r = 0.9999
	p < 0.0001	p < 0.0001	p < 0.0001	p < 0.0001	p < 0.0001	p < 0.0001
CK-MB	r = 0.9998	r = 0.9998	r = 0.9999	r = 0.9997	r = 0.9999	r = 0.9999
	p < 0.0001	p < 0.0001	p < 0.0001	p < 0.0001	p < 0.0001	p < 0.0001

r = Spearman rank correlation coefficients, p = p-value.

VTO, vascular total occlusion group; VIO, vascular incomplete occlusion group.

**Figure 3:**
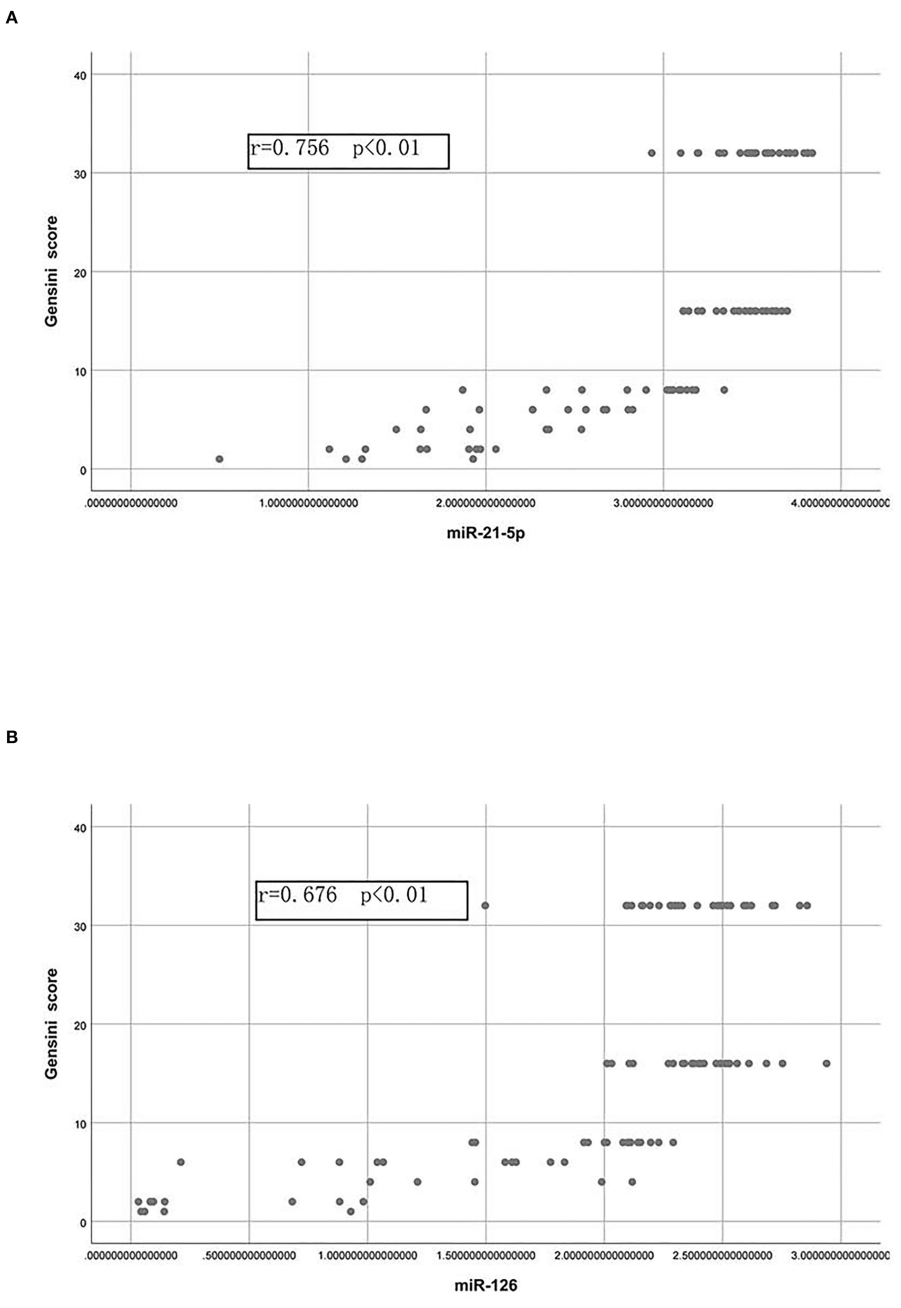
Correlations of the Gensini score with the plasma levels of miR-21-5p and miR-126. **(A)** Pearson correlation between the Gensini score and the levels of miR-21-5p. **(B)** Pearson correlation between the Gensini score and the levels of miR-126.

Pearson's correlation analysis was then performed to determine the correlations of the Gensini scores with miR-21-5p and miR-126 (Figures 3A,B). A strong positive correlation was observed between the Gensini score and the miR-21-5p levels and between the Gensini score and the miR-126 levels in all patients.

### The diagnostic accuracy of miR-21-5p and miR-126

**Figure 4:**
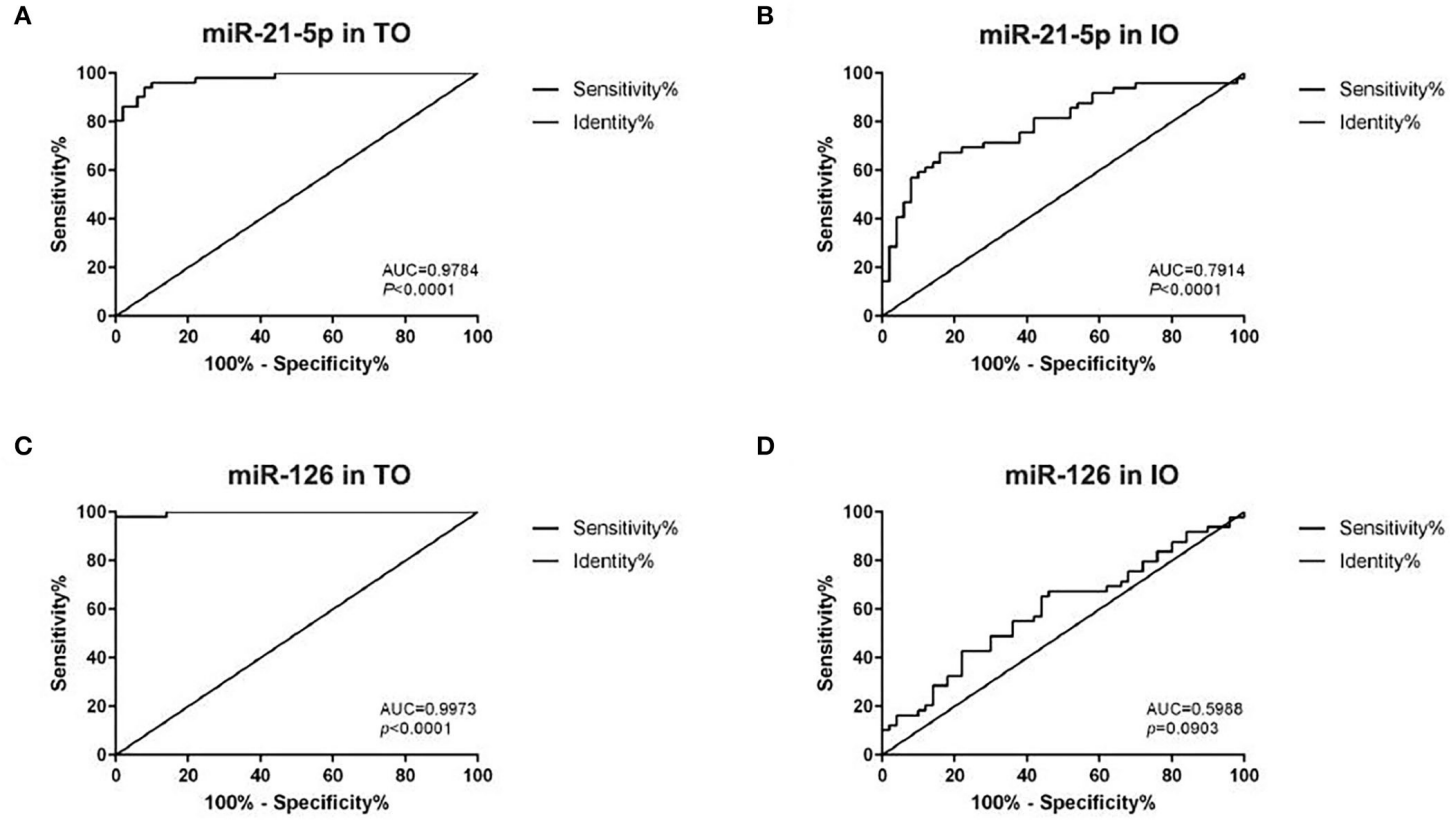
Receiver operating characteristic analysis of miR-21-5p and miR-126. The areas under the curves are 0.9784 (95% CI: 0.9558–1.001, *p* < 0.0001) for miR-21-5p in the infarct-related artery total occlusion (IR-ATO) acute myocardial infarction (AMI) group, **(A)** 0.7914 (95% CI: 0.7014–0.8814, *p* < 0.0001) for miR-21-5p in the IR blood-vessel recanalization (BVR) AMI group, **(B)** 0.9973 (95% confidence interval [CI]: 0.9913–1.003, *p* < 0.0001) for miR-126 in the IR-ATO AMI group, **(C)** 0.5988 (95% CI: 0.4866–0.7110, *p* = 0.0903) for miR-126 in the IR-BVR AMI group, and **(D)** VO = IR-ATO group, and VIO = IR-BVR group.

An analysis of the ROC and AUC was conducted to investigate the diagnostic accuracy of miR-21-5p and miR-126 in patients with AMI and IR-ATO or IR-BVR (Figure 4). The AUC for miR-21-5p was 0.9784 (95% CI: 0.9558–1.001, *p* < 0.0001) in the IR-ATO group and 0.7914 (95% CI: 0.7014–0.8814, *p* < 0.0001) in the IR-BVR group (Figures 4A,B). The AUC for miR-126 was 0.9973 (95% CI: 0.9913–1.003, *p* < 0.0001) in the IR-ATO group and 0.5988 (95% CI: 0.4866–0.7110, *p* = 0.0903) in the IR-BVR group (Figures 4C,D).

## Discussion

It has been demonstrated that a reduction in myocardial blood perfusion and oxygen supply caused by coronary artery occlusion induces myocardial infarction (MI) (17). The presenting symptom of MI is chest pain or discomfort described as pain, pressure, tightness, heaviness, burning, or a squeezing or crushing sensation lasting 20 min or longer (18). In addition, the major pathological changes of MI include ischemia, inflammation, cardiomyocyte apoptosis, cardiac fibrosis, myocardial extracellular matrix lysis, and contractile dysfunction (19). Under the guidelines of the American College of Cardiology/American Heart Association, AMI covers three disorders: unstable angina, non-STEMI (formerly called non-Q wave MI), and STEMI (formerly called Q wave MI) (20). This article focuses on STEMI.

Circulating miRs are promising novel biomarkers for the diagnosis and prognosis of different types of cardiovascular diseases (21). Jaguszewski et al. (22) reported a signature of four circulating miRs as a robust biomarker to distinguish tako-tsubo syndrome from patients with AMI, which highlighted distinct characteristics of the miR profile in clinically indistinguishable cardiovascular diseases. Various miRs have been widely studied for their modulation of cardiomyocyte apoptosis, inflammation, angiogenesis, and fibrosis of AMI (23). Of these, miR-214 is highly expressed in the sera of elderly patients with AMI and may inhibit myocardial cell apoptosis by inhibiting the expression of miR-214 target genes, including the p53-upregulated modulator of apoptosis, PTEN, Bcl-2-associated X, and caspase 7 (24). The overexpression of miR-488-3p suppresses AMI-induced cardiomyocyte apoptosis by targeting zinc finger protein 791 (25). The downregulation of miR-130 expression alleviates AMI by targeting peroxisome proliferator-activated receptor-γ, which has a cardioprotective effect by inhibiting nuclear factor kappa-light-chain-enhancer of activated B cells-mediated inflammation and transforming growth factor beta 1-mediated fibrosis (26). The overexpression of miR-210 promotes angiogenesis in AMI by stimulating hepatocyte growth factor expression and inducing improved left ventricular remodeling (27). Furthermore, accumulated evidence has revealed the effectiveness of circulating miRs used as candidate biomarkers for the prognostic and diagnostic evaluation of AMI. Of these, miR-184 presents as a potential dynamic biomarker before and after percutaneous coronary intervention treatment for AMI (28). Peripheral blood miR-124 is accurate for the early diagnosis of AMI (29), and circulating miR-1 within 3 h of acute chest pain has potential prognostic and diagnostic value for AMI (30).

Both miR-126 and miR-21 are highly implicated in ischemic heart disease. Overexpression of miR-126 has been shown to protect hypoxic-reoxygenation-exposed human umbilical vein endothelial cellular injury (31). Jansen et al. reported that miR-126 correlates with ischemic heart disease in patients with coronary artery disease (32). Elevated miR-21 can be detected in patients with acute coronary syndrome who showed symptom onset within 3 h (33). The intracellular effects of miR-21 have also been shown to improve cardiac function post-AMI (34). In the present study, we delineated the difference in expression of miR-21-5p and miR-126 in patients with IR-ATO and IR-BVR. Both in the IR-ATO group and the vascular-incomplete group, circulating miR-21-5p was significantly upregulated when compared with the control group. In addition, when compared with the control group, the expression of miR-126 in patients with IR-ATO was higher, but no significant difference was found in patients with IR-BVR. The serum levels of cTn-I and CK-MB in patients with IR-ATO and IR-BVR were significantly higher than in the control group, and the expressions of miR-21-5p and miR-126 were strongly correlated with each other and with the levels of cTn-I, the levels of CK-MB, and the Gensini score separately. When compared with the control group, the ROC analysis revealed that the AUC for miR-21-5p and miR-126 in the IR-ATO group was significantly higher, miR-21-5p in the IR-BVR group was significantly higher, and miR-21-5p in the IR-BVR group showed no significant difference. This suggests that miR-21-5p is more accurate for diagnosing both IR-BVR and IR-ATO, and miR-126 is more accurate for diagnosing IR-ATO than IR-BVR.

The kinetics analysis of troponin showed that the concentration of troponin increased within 8 h after symptom onset in patients with acute myocardial infarction (35). The kinetics of circulating miRs during heart attack remains poorly understood. Few miR kinetics studies have been performed. We found a recent study showing that miR-1 and miR-133b were enriched in circulating microparticles (within a couple of hours) following primary percutaneous coronary intervention in a monophasic or biphasic pattern in patients with myocardial infarction (36). Whether miRs share the same kinetics patterns during heart attack warrants further investigation.

Despite these findings, the small sample size limits the usefulness of miR-21-5p and miR-126 as diagnostic biomarkers for patients with AMI and IR-ATO or IR-BVR. Large clinical studies are needed to confirm the results. In addition, we used only one housekeeping gene (U6) for analyzing the qPCR results. The use of other housekeeping genes will be needed to validate the current findings.

## Conclusion

The present study found that miR-21-5p may be a promising diagnostic marker for patients with IR-ATO AMI and vascular-incomplete AMI, and miR-126 may be used for the diagnosis of IR-ATO AMI rather than vascular-incomplete AMI. These findings support further investigations and clinical use of circulating miR-21-5p and miR-126 as diagnostic biomarkers for IR-ATO AMI.

## Data availability statement

The original contributions presented in the study are included in the article/supplementary material, further inquiries can be directed to the corresponding author.

## Ethics statement

The studies involving human participants were reviewed and approved by Ethics Committee of Shanxi DAYI Hospital (SBQLL-2017-035). The patients/participants provided their written informed consent to participate in this study.

## Author contributions

Conception and design of the research: X-lM and X-wL. Critical revision of the manuscript for intellectual content and obtaining financing: X-wL. Writing of the manuscript: X-lM. Statistical analysis: Z-jZ. Analysis and interpretation of the data: Y-pG. Acquisition of data: D-jH, ZX, and TL. All authors read and approved the final draft.

## Funding

This work was funded by Shanxi Natural Science Foundation (Grant no. 201701D121148).

## Conflict of interest

The authors declare that the research was conducted in the absence of any commercial or financial relationships that could be construed as a potential conflict of interest.

## Publisher's note

All claims expressed in this article are solely those of the authors and do not necessarily represent those of their affiliated organizations, or those of the publisher, the editors and the reviewers. Any product that may be evaluated in this article, or claim that may be made by its manufacturer, is not guaranteed or endorsed by the publisher.
